# Implantation of autogenic and decellularized xenogenic grafts for tissue repair in experiment

**DOI:** 10.3389/fcell.2026.1787725

**Published:** 2026-02-27

**Authors:** Alexander V. Pechersky, Viktor I. Pechersky, Ilya A. Barsuk, Vladimir N. Vilyaninov, Viktor N. Alexandrov, Vladimir F. Semiglazov

**Affiliations:** 1 Medical Center, St. Petersburg, Russia; 2 Retired, St. Petersburg, Russia; 3 Scientific Research Center, Military Medical Academy named after S.M. Kirov, St. Petersburg, Russia; 4 Blood and Tissue Center, Military Medical Academy named after S.M. Kirov, St. Petersburg, Russia; 5 Scientific Department of Breast Tumors, National Medical Research Center of Oncology named after N.N. Petrov, St. Petersburg, Russia

**Keywords:** autogenic and decellularized xenogenic grafts, innate and acquired immunity, pluripotent stem cells, regeneration, tissue damage, tissue-specific antigens

## Abstract

**Introduction:**

With extensive tissue damage, the body is unable to restore their integrity on its own. Implantation of autogenic and decellularized xenogenic grafts opens up new possibilities for regeneration of damaged corresponding tissues.

**Methods:**

The pilot experimental study was conducted on a model of healing of grade III B skin burn wounds in Wistar rats. After removal of the necrotized tissues, autogenous and decellularized xenogenic grafts were implanted into the blood-supplying tissues of the burn wounds.

**Results:**

The pilot experimental study showed that implantation of autogenic and decellularized xenogenic grafts in the experimental zone led to the formation of multiple regeneration sites, almost ten times higher than the marginal epithelialization of the control zone. The proportion of epithelialization of the experimental zone initiated by the installed grafts was more than 90%, and the proportion of marginal epithelialization of the control zone was less than 10%. The completion of epithelialization of skin burn wounds with a predominance of epithelialization of the experimental zone led to the healing of burn wounds. The tightening of the wound edges by scar tissue was minimal.

**Conclusion:**

Implantation of autogenic or decellularized xenogenic grafts can potentially be used to repair any tissues after their damage or disease. The results obtained are preliminary, requiring verification on a wider sample of experimental animals. The use of this methodology to repair tissues with a more complex structure than the skin, for increase the functioning of the parenchyma of various organs requires further study.

## 1 Introduction

With extensive tissue damage, the body is not able to restore their integrity on its own. To repair extensive tissue damage, decellularized connective tissue (stromal) matrices made from tissues similar to the damaged ones must be transplanted to ensure specific differentiation of migrated pluripotent stem cells (Pechersky et al., 2008; Pechersky et al., 2016a). The use of mesenchymal stem cells (Carlos Rodriguez-Merchan, 2022) and gels made from extracellular matrix (Deng et al., 2025) is effective for small defects, but cannot ensure the restoration of extensive tissue damage. Mesenchymal stem cells (Dominici et al., 2006) – the precursors of fibroblasts and their derivatives: adipocytes, chondrocytes, and osteocytes (Pechersky, 2016), are unable to form a specific three-dimensional connective tissue matrix of damaged tissue after transplantation without the influence of the cellular environment that died at the site of injury. Similarly, gels prepared from the extracellular matrix of tissues other than damaged ones contain a set of non-specific cellular growth factors that are unable to provide specific differentiation of migrated pluripotent stem cells into cells of damaged tissues. Currently, there is no generally recognized consensus on the decellularization procedure, which allows preserving the molecular composition of the allogeneic or xenogenic extracellular matrix, and the method of its recellularization (Gentile et al., 2020). The study of new methods of tissue regeneration in their injuries and diseases, including those leading to a decrease in the functioning parenchyma of organs formed from them, is an actual task facing fundamental and applied medicine.

## 2 Materials and methods

The aim of the pilot experimental study was to proof of concept of the formation of multiple regeneration sites during implantation of autogenic and decellularized xenogenic grafts into blood-supplied tissues in cases of extensive damage. A grade III B skin burn injury in experimental animals was used as a model of extensive tissue damage. The study was conducted on two Wistar rats (males aged 4 months), each of which received a grade III B skin burn under general anesthesia in two areas to the left and right of the withers. For the thermal burn, a metal cylinder with a diameter of 5 cm was used, heated to a temperature of 100° C. The exposure was 30 s. On day 3, necrotic tissues were removed and grafts were inserted with micro tweezer into cavities formed by a micro scalpel in the underlying tissues, which retained blood supply and viability. Grafts of 2 × 2 mm made of decellularized and lyophilized xenogenic (pig) skin (Xenoderm biological coating approved for clinical use) were installed in the burn area on the right side. The quality of decellurization of the Xenoderm biological coating is confirmed by the manufacturer’s certificate, which excludes the participation of donor (pig) cells in the epithelization of the matrix. The biological coating of Xenoderm, as a stromal matrix of xenogenic skin, was only able to direct the differentiation of migrated own pluripotent stem cells of the experimental animal. Grafts with a diameter of 1 mm were placed in the burn area on the left side, which included all layers of the auto skin of the laboratory animal, harvested immediately before implantation from a nearby donor skin area using the graft extraction technique for hair follicle transplantation.

The very fact of epithelialization initiated by established autogenic and decellularized xenogenic grafts was evaluated, as well as the ratio of the epithelialization area from the graft sites (which formed the experimental zone) and marginal epithelialization (which formed the control zone). The comparison of the epithelialization of the experimental and control zones of one wound of one animal ensured the objectivity of the evaluation of the experimental results, regardless of the features of the wound process in different animals.

## 3 Results

**FIGURE 1:**
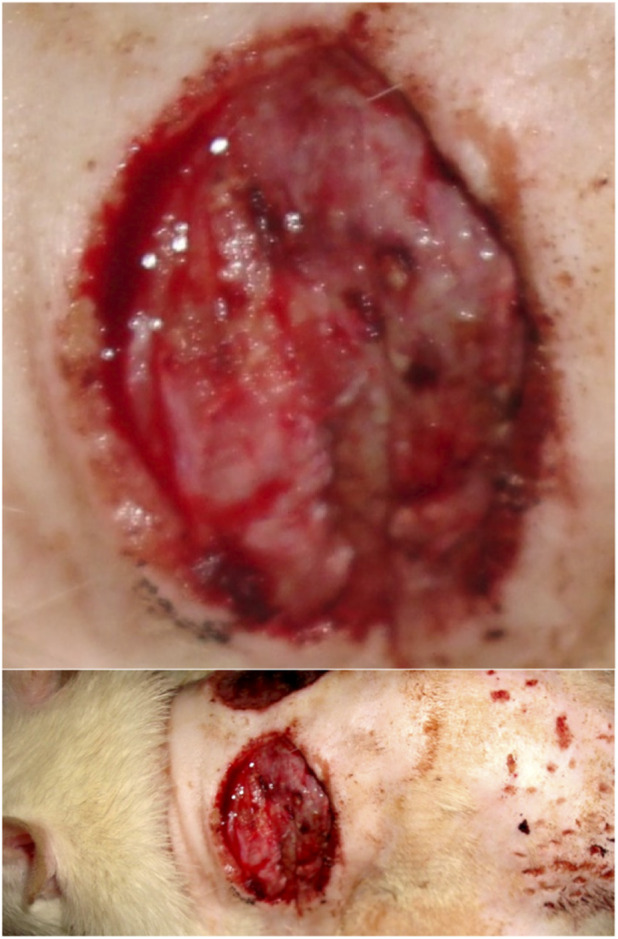
Animal No. 1 (burn area and general appearance + donor site), 1st day after graft implantation (4th day after burn injury). The burn area on the left side with implanted auto filament grafts obtained from a nearby donor skin area.

**FIGURE 2:**
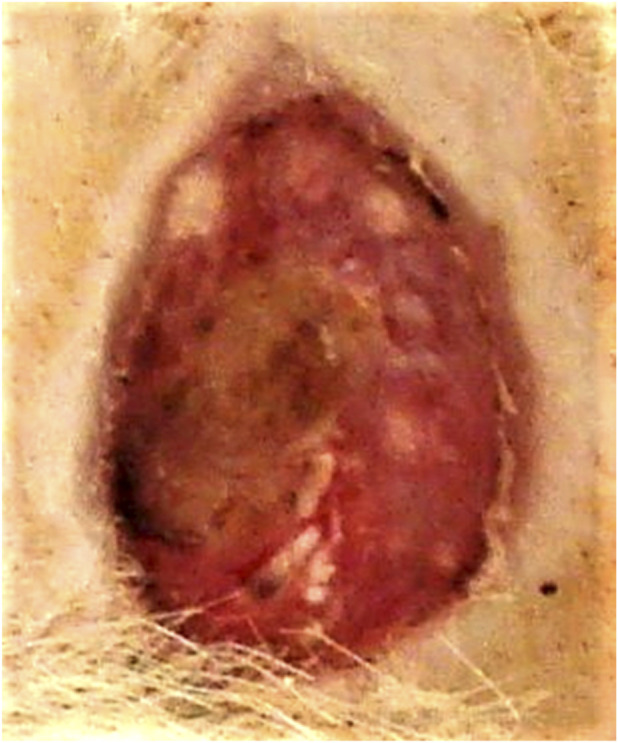
Animal No. 1, 9th day after graft implantation (12th day after burn injury). The burn area on the left side with implanted auto filament grafts obtained from a nearby donor skin area.

**FIGURE 3:**
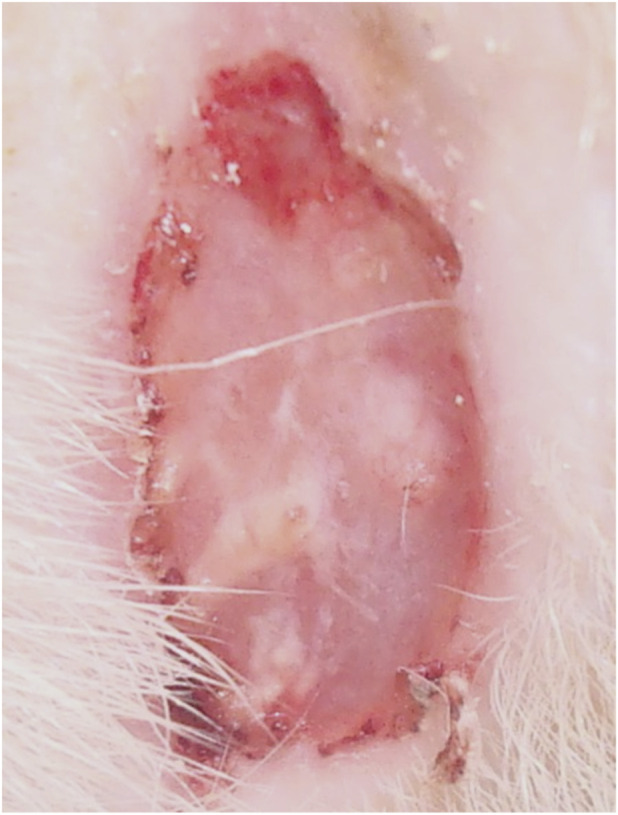
Animal No. 1, 18th day after graft implantation (21st day after burn injury). The burn area on the left side with implanted auto filament grafts obtained from a nearby donor skin area.

**FIGURE 4:**
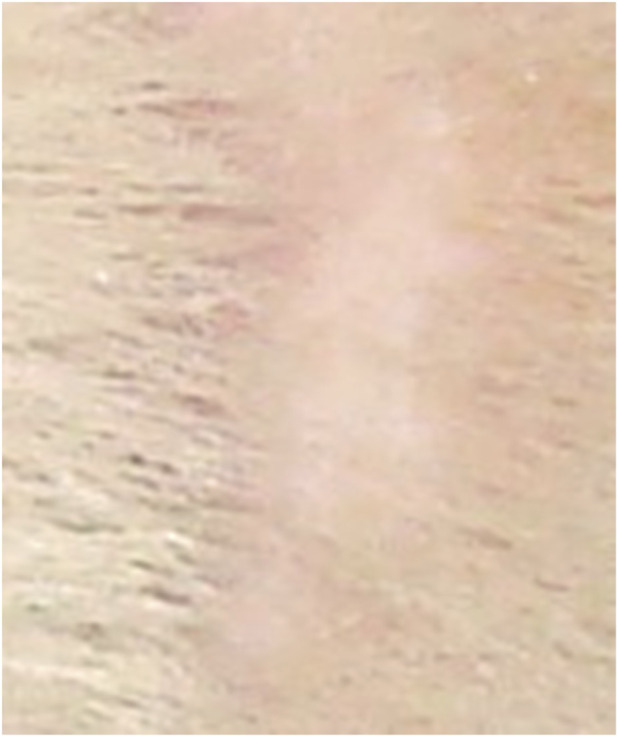
Animal No. 1, 26th day after graft implantation (29th day after burn injury). The burn area on the left side with implanted auto filament grafts obtained from a nearby donor skin area.

**FIGURE 5:**
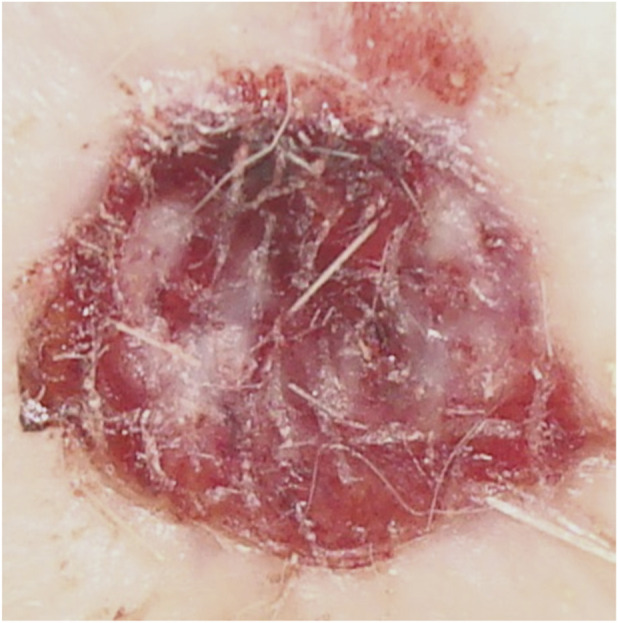
Animal No. 2, 12th day after graft implantation (15th day after burn injury). The burn area on the left side with implanted auto filament grafts obtained from a nearby donor skin area.

**FIGURE 6:**
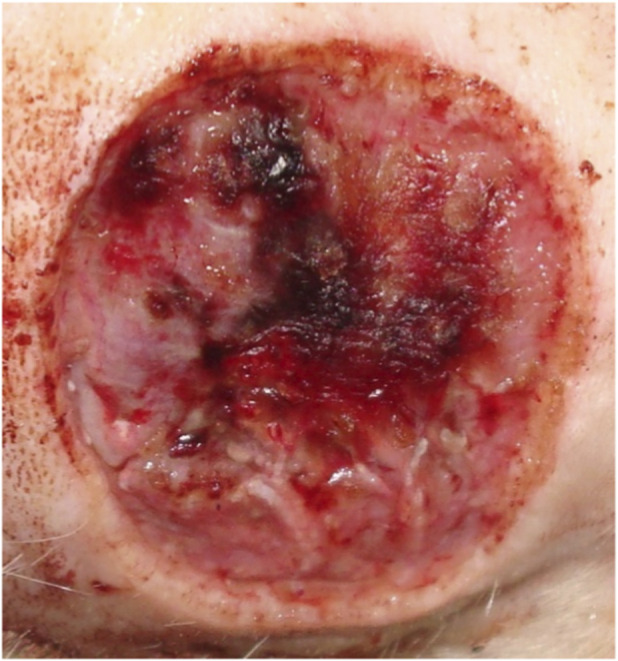
Animal No. 1, 1st day after graft implantation (4th day after burn injury). The burn area on the right side with implanted grafts of decellularized and lyophilized xenogenic (pig) skin (Xenoderm).

**FIGURE 7:**
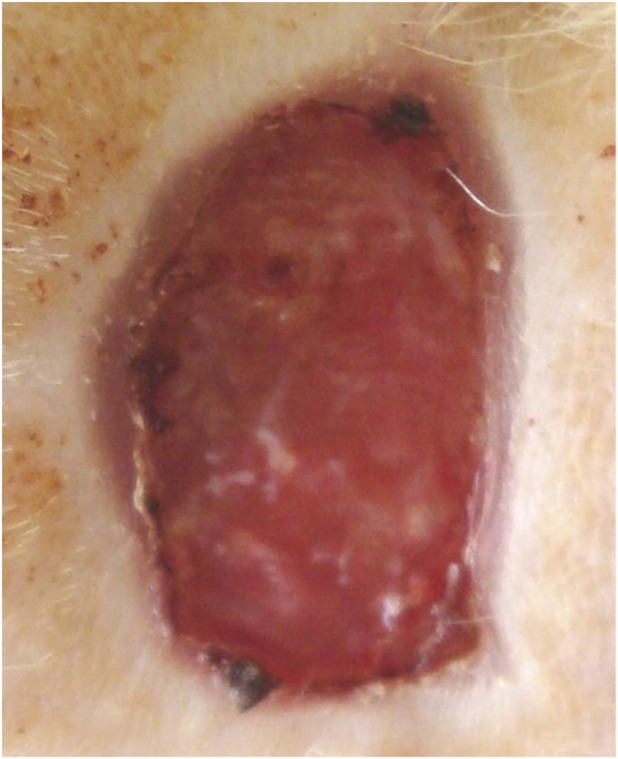
Animal No. 1, 9th day after graft implantation (12th day after burn injury). The burn area on the right side with implanted grafts of decellularized and lyophilized xenogenic (pig) skin (Xenoderm).

**FIGURE 8:**
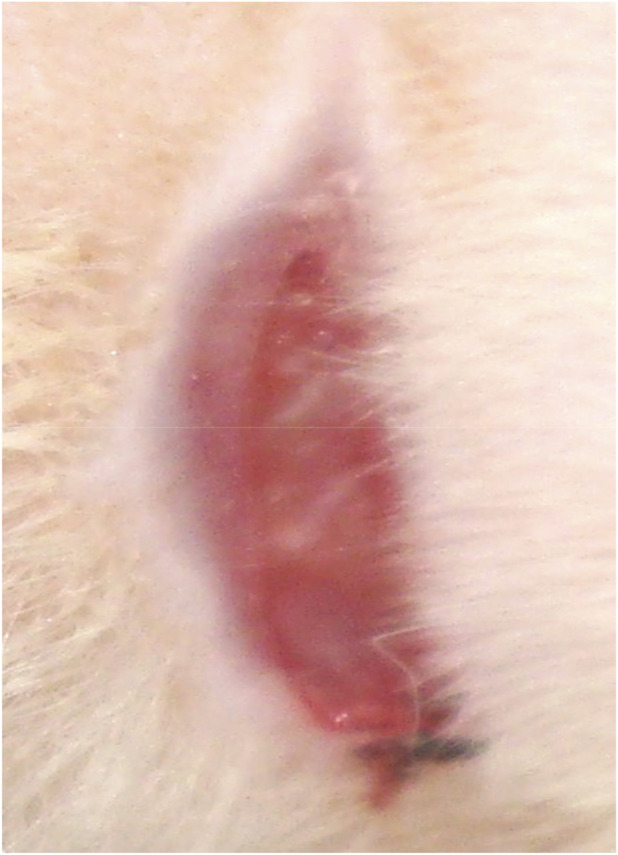
Animal No. 1, 18th day after graft implantation (21st day after burn injury). The burn area on the right side with implanted grafts of decellularized and lyophilized xenogenic (pig) skin (Xenoderm).

**FIGURE 9:**
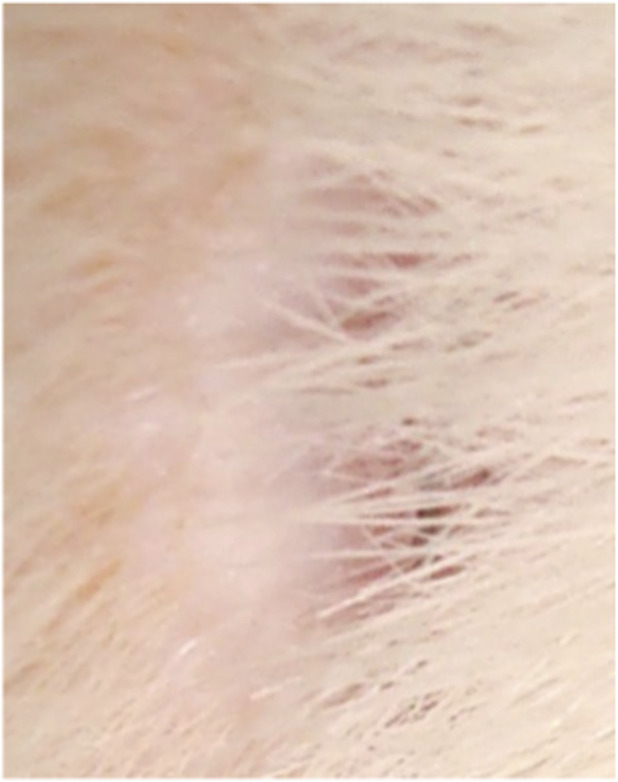
Animal No. 1, 26th day after graft implantation (29th day after burn injury). The burn area on the right side with implanted grafts of decellularized and lyophilized xenogenic (pig) skin (Xenoderm).

**FIGURE 10:**
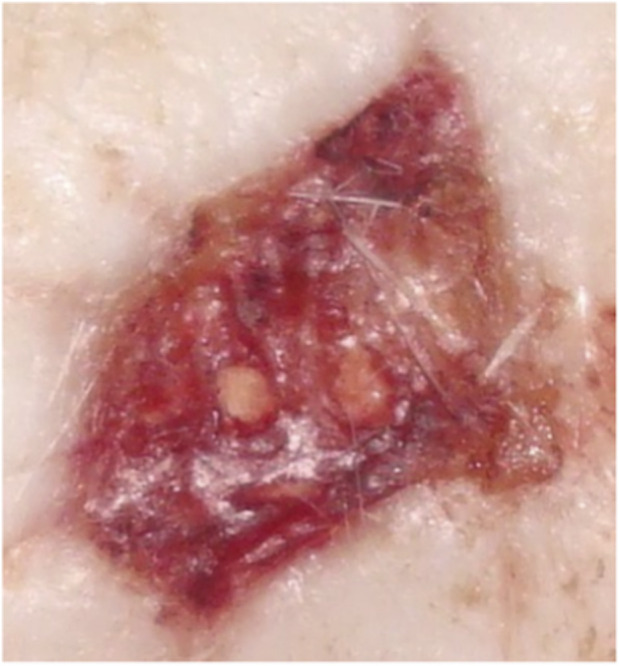
Animal No. 2, 12th day after graft implantation (15th day after burn injury). The burn area on the right side with implanted grafts of decellularized and lyophilized xenogenic (pig) skin (Xenoderm).

After implantation on days 9–18 (days 12–21 after thermal injury), autogenous and decellularized xenogenic skin grafts became multiple centers of epithelialization in the experimental zone, which significantly prevailed over the marginal epithelialization of the control zone. The proportion of epithelialization initiated by established grafts was more than 90%, and the proportion of marginal epithelialization was less than 10%. Epithelialization of skin burn wounds with an almost tenfold predominance of epithelization in the experimental zone resulted in wound healing in both areas with autogenic grafts and decellularized xenogenic grafts. The tightening of the wound edges by scar tissue was minimal. The intensity of epithelialization in the experimental zone of both experimental animals was higher on the left, on the implantation side of autogenic skin grafts (Figures 1–10). By day 26 (29 days after thermal injury), hair growth began at the site of the completed epithelialization of burn wounds. Histological examination confirmed a grade III B burn - necrosis of all skin layers and additional skin elements (hair follicles and sebaceous glands).

## 4 Discussion

An experimental study using the example of epithelialization of animal skin after a grade III B burn injury demonstrated the possibility of restoring damaged tissues through low-traumatic and technically simple implantation of autogenic or decellularized xenogenic grafts forming multiple regeneration sites (Figures 1–10). Since the restoration of damaged tissues depends on the ratio of regeneration and fibrosis processes, the predominance of epithelialization (90% of which was initiated by grafts installed in experimental zone) led to minimal tightening of the wound edges by scar tissue and wound healing. The onset of hair growth at the site of burn wounds by day 26 (29 days after thermal injury) was evidence of the restoration of secondary skin elements, hair follicles, during epithelialization (which occurred mainly from graft sites). When scar tissue replaces all layers of necrotic skin with a grade III B skin burn, skin repair, including hair follicles and hair growth, does not occur.

The similarity of the results of epithelialization in both experimental animals with an almost tenfold predominance of epithelialization in experimental zone with established grafts indicates a minimal probability of accidental data acquisition. The histological examination confirmed a grade III B burn - necrosis of all skin layers, which excluded the possibility of subsequent regeneration from cells of additional skin elements (hair follicles and sebaceous glands).

Unlike the transplantation of the donor stromal base of the entire parenchymal organ, which requires connecting its vessels to the recipient’s bloodstream to ensure the supply of a sufficient number of pluripotent stem cells (Pechersky et al., 2008; Pechersky et al., 2016a; Pechersky et al., 2019), when using autogenic or decellularized xenogenic grafts, their implantation into the recipient’s blood-supplied tissues is sufficient.

The preferred explanation for the mechanism of directed migration of pluripotent stem cells to implanted autogenic and decellularized xenogenic grafts is the involvement of the immune system in it, described in the previously proposed immune concept of regeneration (Pechersky et al., 2008; Pechersky et al., 2016a; Pechersky et al., 2019). The direction of differentiation of migrated pluripotent stem cells is determined by cellular growth factors of connective tissue located on glycoproteins of intercellular matrix and basement membranes. The sequence and composition of cellular growth factors of glycoproteins of the intercellular matrix and basement membranes form a unique differentiation code of migrated pluripotent stem cells into cells of the corresponding tissues formed during the implementation of the development program (Pechersky et al., 2008; Pechersky et al., 2016a; Pechersky et al., 2019; Alberts et al., 1994). After removal by proteolytic enzymes of parenchymal cells of donor tissues or organs carrying peptide copies of tissue-specific antigens associated with MHC (major histocompatibility complex) class I dimers (HLA in leukocytes) and transplantation of the remaining stromal base, the recipient’s tissue structure is restored due to migration of his own pluripotent stem cells and their differentiation into cells of this tissue or organ (Pechersky et al., 2008; Pechersky et al., 2016a; Pechersky et al., 2019).

The stromal base of allogeneic and xenogenic tissues and organs, consisting of an intercellular matrix and basement membranes, has low immunogenicity (Alberts et al., 1994), which is insufficient to initiate differentiation of T-helper (Th) into T-helper 1 (Th 1), forming tissue-specific receptors of cytotoxic T-cells, or into T-helper 2 (Th2), regulating the formation of antibody-producing B-cells. The low immunogenicity of the exposed collagen of the intercellular matrix and basement membranes does not interfere with platelet aggregation producing vasoactive amines and chemoattractants (Roitt et al., 2000). The latter attract antigen-presenting cells (macrophages, dendritic cells, and others) that initiate the differentiation of T-helper (Th) into T-helper 1 (Th 1), which form tissue-specific receptors in stem cells (Pechersky et al., 2008; Pechersky et al., 2016a; Pechersky et al., 2019). Subsequent directed migration of the recipient’s own committed pluripotent stem cells with formed tissue-specific receptors provides regeneration - restoration of the donor stromal base into a full-fledged tissue or organ of the recipient (Pechersky et al., 2008; Pechersky et al., 2016a; Pechersky et al., 2019; Pechersky, 2016; Pechersky et al., 2016b). The leading regenerative function (rather than the protective function) of acquired immunity (Pechersky et al., 2008; Pechersky et al., 2016a; Pechersky et al., 2019) is evidenced by the binding of MHC class II molecules of antigen-presenting cells and T-helper (Th) to 99% of the peptide copies of autoantigens and only to 1% of the peptide copies of foreign antigens (Pechersky et al., 2008). Presentation to T-helper of 99% of the peptide copies of autoantigens of dead old and damaged cells are completed by the formation of the same proportion of complementary receptors to autoantigens in pluripotent stem cells (Pechersky et al., 2008; Pechersky et al., 2016a; Pechersky et al., 2019). Directed migration of pluripotent stem cells is impossible without the formation of tissue-specific receptors. Naturally, local administration of pluripotent stem cells does not lead to their differentiation into tissues cells of the injection site; after administration, pluripotent stem cells, lacking tissue-specific receptors, migrate from the injection site.

The higher intensity of epithelialization of the burn wound, obtained by using autogenic grafts (Figures 1–5), indicates the need to improve the production technology of xenogenic decellularized stromal matrices. During their production, it is necessary to preserve their cellular growth factors the unique code of the composition and sequence of which directs the differentiation of migrated pluripotent stem cells into cells of certain tissues from which these matrices are prepared.

Despite the low immunogenicity of xenogenic connective tissue matrices, tissues and organs for their manufacture should be taken from animal donors with a blood type similar to human blood type 0 (I) (having an antigen similar to human factor H of blood type 0 (I)), as universal donors without antigens, interacting with aglutinins α and β. Because blood group antigens are present not only on red blood cells, but also in various tissues (Kosyakov, 1974), this methodology will minimize the risk of developing innate immunity reactions and secondary developing acquired immunity reactions (Pechersky et al., 2019).

## 5 Conclusion

Implantation of autogenic and decellularized xenogenic grafts forming multiple regeneration sites opens up new potential opportunities for tissue repair after their damage or disease. In some cases, this technology may become an alternative for more complex and traumatic transplantation of tissues and organs. The results of the pilot study are preliminary, requiring verification on a wider sample of experimental animals. The use of this methodology to repair tissues with a more complex structure than skin to increase the functioning parenchyma of various organs requires further study.

## Data Availability

The original contributions presented in the study are included in the article/supplementary material, further inquiries can be directed to the corresponding author.
